# Welcome to Stem Cell Reports

**DOI:** 10.1016/j.stemcr.2013.05.003

**Published:** 2013-06-04

**Authors:** Christine Mummery, Yvonne Fischer, Atie Gathier

**Affiliations:** Editor in Chief, *ISSCR*; Managing Editor, *ISSCR*; Editorial Assistant, *ISSCR*

## Main Text

On behalf of the International Society for Stem Cell Research (ISSCR) and the *Stem Cell Reports*’ editorial team, we would like to extend a warm welcome to you and present the inaugural issue of our journal, *Stem Cell Reports*.

**Figure:**
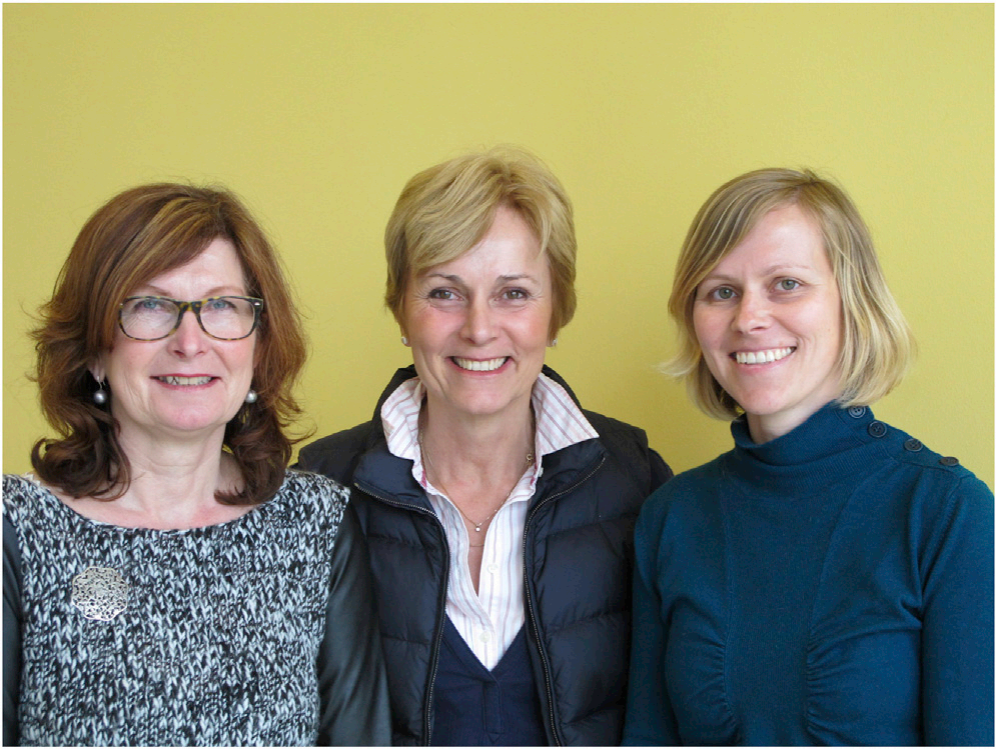
Stem Cell Reports Editorial Team Atie Gathier (Editorial Assistant, left), Christine Mummery (Editor in Chief, center), Yvonne Fischer (Managing Editor, right).

*Stem Cell Reports* is the ISSCR’s new journal, a highly visible, open access forum, accelerating the speed with which advances and new ideas are shared and expanded. The mission of our new journal is to deliver significant, well-documented findings to the stem cell research community in a timely manner. *Stem Cell Reports* focuses on shorter, single-point reports in addition to full length articles, and offers a fair peer-review process supervised by leading scientists in the field.

It is our particular interest to cover all areas of stem cell research comprehensively and we are delighted to have received manuscripts reporting research on a wide range of stem and progenitor cell types from varied species and model systems. The current issue presents examples from many of our interest areas: embryonic stem cells and the roles of wnt signaling in determining fate, the conversion of primordial germ cells to pluripotency, studies of telomere length in human mammary gland progenitors, the identity of mouse interfollicular epidermis progenitors, and the role of SOX2-positive neural crest progenitors in skin repair. Furthermore, we have two “Resource” articles this month, one describing tools for studying transgenesis in axolotl and the other, a compilation of imprinted loci in human induced pluripotent stem cells. The lineup for the second issue is already well advanced and we will follow with monthly issues that will continue to cover a wide range of research from developmental biology, stem and progenitors cells, fate determination, and the genomics and epigenetics of these systems to disease models, tissue engineering, and regenerative medicine.

The inaugural issue contains examples of all of our research article formats: Reports, full-length Articles, and Resources. In addition, it features an historical review, perhaps one of the most comprehensive you will ever read, of the science that formed the background to the shared Nobel Prize for Sir John Gurdon and Shinya Yamanaka, ISSCR president 2012–2013.

Starting a new journal is both exciting and challenging. A successful launch relies on scientists submitting, reviewing, and editing manuscripts—taking a “leap of faith” and really committing their time to making it a success. For *Stem Cell Reports,* this support has reached far beyond our expectations: we have had nearly 100 submissions since the first call for papers in December 2012, many of high quality and some real “gems”; reviewers have been responsive and have invested their energy as altruistically as for established journals; and the ISSCR’s Board of Directors, ISSCR members, and the *Stem Cell Reports* Editorial Board have both reviewed and submitted manuscripts with exceptional loyalty. This first issue is a testimony to their efforts and also to those of the ISSCR’s Publications Committee, which had the foresight to initiate a society stem cell journal and to identify the particular niche *Stem Cell Reports* could fill. We have been delighted by the support of our publisher, Cell Press, which has guided us through the extremely tight timelines necessary for this inaugural issue to be ready for the ISSCR 11^th^ Annual Meeting in Boston. We are grateful to all authors and referees, and in particular we appreciate their patience and understanding as we, the editorial team, have learned the behind-the-scenes system and occasionally pressed the wrong buttons.

We would also like to take this opportunity to introduce the rest of our editorial team. An important aspect of *Stem Cell Reports* is that the editorial leadership is provided by scientists active in the field, and we are delighted to introduce a truly international and scientifically renowned group of Associate Editors: Nissim Benvenisty, M.D., Ph.D. (Hebrew University, Israel), Thomas Graf, Ph.D. (Center for Genomic Regulation, Spain), Hideyuki Okano, M.D., Ph.D. (Keio University School of Medicine, Japan), and David Scadden, M.D., Ph.D. (Massachusetts General Hospital/Harvard University, USA). We look forward to hearing about your science and encourage you to approach us at meetings to ask us more about *Stem Cell Reports*.

We hope you will enjoy reading this issue as much as we have enjoyed producing it. We hope, too, that it will inspire you to send us your next manuscript.

